# Trends in baseline CD4 cell counts and risk factors for late antiretroviral therapy initiation among HIV-positive patients in Shanghai, a retrospective cross-sectional study

**DOI:** 10.1186/s12879-017-2398-5

**Published:** 2017-04-19

**Authors:** Jianjun Sun, Li Liu, Jiayin Shen, Panpan Chen, Hongzhou Lu

**Affiliations:** 1 Department of Infectious Diseases, Shanghai Public Health Clinical Center, Fudan University, Shanghai, 201508 China; 2 Pudong New Area Center for Disease Control and Prevention, Shanghai, 200136 China; 3 Department of Infectious Diseases, Huashan Hospital Affiliated to Fudan University, Shanghai, 200040 China; 4 Department of Internal Medicine, Shanghai Medical College, Fudan University, Shanghai, 200032 China

**Keywords:** HIV infection, Baseline, CD4 cell counts, Trends, Late ART initiation, Risk factors

## Abstract

**Backgrounds:**

There are few studies focus on the factors underlying the late initiation of ART in China. We analyzed the trends in the median CD4 cell counts among different patient groups over time and the risk factors for the late initiation of ART in Shanghai, China.

**Methods:**

A retrospective cross-sectional survey was made in the Department of Infectious Disease of Shanghai Public Health Clinical Center which is a designated diagnosis and treatment center for HIV-positive patients in Shanghai during the period of January 1st, 2008--June 30th, 2014. Late ART initiation was defined as a CD4 cell count <200 cells/mm^3^ or having a clinical AIDS diagnosis prior to ART initiation. Trends in the median CD4 cell count at ART initiation and the proportion of late ART initiation by year were evaluated using Spearman’s correlations and Chi-squared methods, respectively. We used a logistic regression model to analyze the risk factors for late ART initiation. The related factors collected in the multivariate model were the patient’s age, gender, infection routes and marital status.

**Results:**

A total of 3796 patients were analyzed in this study, with a median baseline CD4 cell count of 205 cells/mm^3^ [interquartile range: 75–287]. The median CD4 cell counts of patients initiating ART late increased from 76 cells/mm^3^ in 2008 to 103 cells/mm^3^ in 2014 (*p* < 0.001), and the proportion of late ART initiation decreased from 80% to 45% (*p* < 0.001). The risk factors for late ART initiation were male gender, heterosexual transmission and older age (>30 years) (*p* < 0.001).

**Conclusions:**

Notable improvements were made in the median CD4 cell count at ART initiation and the proportion of late ART initiation from 2008 to 2014. However, persons with high risk of HIV exposure who are male, older even heterosexual orientation should be given more opportunities to receive frequently screening, earlier diagnoses and timely treatment.

**Electronic supplementary material:**

The online version of this article (doi:10.1186/s12879-017-2398-5) contains supplementary material, which is available to authorized users.

## Background

The morbidity and mortality of HIV infection have been sharply decreasing all over the world due to the global implementation of antiretroviral therapy (ART), and now HIV infection is recognized as a chronic disease instead of a deadly one. Ever since China began to establish national HIV prevention and treatment programs in 2003, the morbidity and mortality of AIDS have been reduced significantly [1]. Since the domestic financial resources being allocated to HIV/AIDS were increasing, the ART eligible criteria in China kept changing accordingly. Initially, it was recommended that ART should be initiated when CD4 < 200 cells/mm^3^, and the criteria changed to <350 cells/mm^3^ in 2008 [1]. In 2013, the recommendation to initiate ART at CD4 counts <500/mm^3^ was documented [2] while in this year, ART should be started immediately with the informed consent if HIV infection was determined. At the same time, despite the nationwide scale-up of HIV programs in China over the past decade, the proportion of ART-eligible HIV-positive patients who receive treatment remains low [3, 4]. What even worse is that many HIV-positive people receive late HIV diagnoses [5–7]. Both of these factors weaken the effect of ART among HIV-positive populations [8, 9]. Therefore, the vital steps to maximize the efficacy of ART for HIV infected patients would be expanding screening of HIV antibody and initiating ART as soon as possible when the patients meet the criteria [10]. With the recommendation of expanding ART to all HIV-positive people [11–13], a delay in diagnosis and referral could definitely weaken the effect of expanding the ART program. However, considering the imbalance in the development of the regional economy, the resources for HIV prevention and treatment in Shanghai are more readily available than in the smaller counties in China. Once patients in Shanghai received an HIV screening test and the test was confirmed by Western Blot test, the local CDC would contact the infected patients. Then, the CDC followed up with the HIV-positive patients and performed routine CD4 tests to monitor their immunological status. If the HIV patients met the criteria for ART initiation, the patients were transferred to our clinic and given adherence education, and other preparations were made for the patients to receive ART. The eligible patients started ART within a few days in our clinic. Therefore, the time to initiation in Shanghai was rapid, and patients with more advanced HIV diagnoses received much quicker referral to our clinic. However, we still could receive HIV patients in the late stage of AIDS in our hospital. What factors driving them come to our clinic so late? How to facilitate the screening, diagnosing and ART initiating? There was little data can be found about late ART in China. As we all know that, targeting the HIV-positive population who are most likely to be at risk for being diagnosed or receiving ART late would facilitate the control of the HIV epidemic. Therefore, identifying the factors underlying the current delay of HIV treatment and the risk factors for the late initiation of ART will be helpful to policymakers.

In this study, we conducted a cross-sectional survey of clinical data from the Shanghai Public Health Clinical Center. The trends in the median CD4 cell counts among different patient groups over time and the risk factors for the late initiation of ART were analyzed.

## Methods

### Data collection and definitions

Patients who initiated ART during the period of January 1st, 2008-June 30th, 2014 were collected retrospectively for this survey according to the following criteria: HIV-1 test positive (by Western Blot), aged more than 16 years old, antiretroviral treatment-naïve. Patients with incomplete data, such as a lack of baseline CD4 cell counts, HIV diagnosis date, or information on the time of ART initiation were excluded. Late ART initiation was defined as a CD4 cell count <200 cells/mm^3^ or having a clinical AIDS diagnosis prior to ART initiation. All of the CD4 cell count tests were performed within 30 days prior to treatment initiation. The diagnosis of AIDS was determined by WHO stage IV disease or symptoms. The WHO stage was assessed by the clinicians in the first visit. The patients’ characteristics were recorded at baseline, including age, gender, marital status, and HIV exposure route. The date of HIV diagnosis was recorded according to the referral list from the Shanghai CDC. The time to ART initiation was defined as the time interval between ART initiation and testing positive for Western Blot. We used month-based intervals to assess the length of time from diagnosis to ART initiation. For those whose time to ART initiation was less than 14 days, we defined it as 0.5 month; 15 days to 30 days was defined as 1 month; 31 days to 44 days was defined as 1.5 months; and so forth. In the clinic, some patients reported that no definite infectious routes could be determined, and we identified those patients as having an undetermined route of transmission. All of the related clinical data underlying these results are listed in Additional file 1.

### Data analyses

Continuous variables were described using the median and interquartile range (IQR), while categorical variables were described by percentages. The Chi-square test was used for categorical variables, and the Mann-Whitney test was used for continuous variables. We built a logistic regression model to analyze the risk factors for late ART initiation. The predictors included in the multivariate model were selected based on a significance level of *p* < 0.1 in the univariate analyses. The confounding factors retained in the multivariate model were the patient’s age, gender, calendar year of ART initiation, infection routes and marital status. Trends in the median CD4 cell count at ART initiation and the proportion of late ART initiation by year were evaluated using Spearman’s correlations and Chi-squared methods, respectively. All hypothesis testing was 2-sided with a level of α = 0.05. Data analysis was conducted using IBM SPSS version 19.0 (IBM SPSS, Inc., Armonk, NY, USA), and the figures were constructed using GraphPad Prism version 5.0.

## Results

### Demographics of late and non-late ART initiation groups

**Fig. 1:**
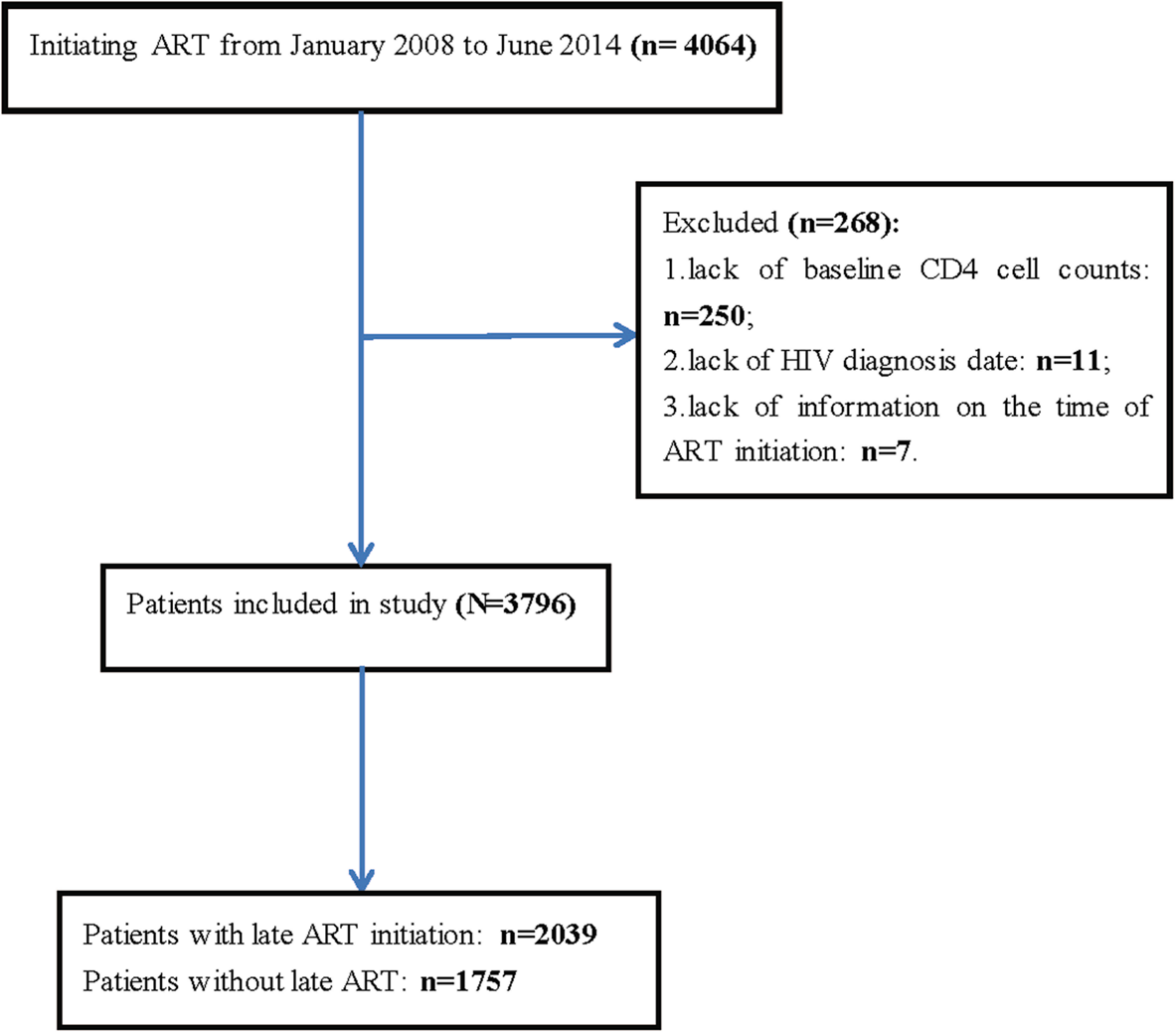
The flow chart of participants selection

A total of 3796 patients were selected, and most of them were male (91%). See the participants selection flow chart in Fig. 1. The age range among the patients initiating ART late was 16 to 87 years old, while the range was 18 to 88 years old in the non-late ART initiation group. There were 614 (16.2%) patients who were infected by undetermined routes. The time to ART initiation ranged from 0.5 to 94.5 months in the late ART initiation group, while this range was 0.5 to 92.5 months among the non-late treatment group. In addition to the WHO stages (not including stage IV) and gender, the other demographics, such as age and marital status, and clinical data, such as the CD4 cell counts and infection routes, were all significantly different between the late ART initiation patients and the non-late ART group (*P* < 0.0001). Notably, the median time to ART initiation in the late ART group was shorter than the non-late initiation group, and this difference was significant (*P* < 0.0001). More details are listed in Table 1.

**Table 1:** Analysis of the demographics and clinical data between late ART initiation and non-late ART initiation groups

Characteristics	Total Patients (*N* = 3796)	Patients with late ART initiation (*N* = 2039)	Patients without late ART (*N* = 1757)	*P*-value
Age (Median, IQR)	35(29--48)	38(30--50)	33(27--44)	< 0.0001^#^
Age stratification				< 0.0001
≤ 30	1273(33.5)	567(28)	706(40.2)	
31–40	1058(27.9)	546(27)	512(29.1)	
41–50	704(18.6)	434(21)	270(15.4)	
≥ 51	761(20)	492(24)	269(15.3)	
Gender				0.10
Male	3457(91)	1872(92)	1585(90.2)	
Female	339(9)	167(8)	172(9.8)	
Marital status				< 0.0001
Married or live together	1583(42)	945(46.3)	638(36)	
Single	1861(49)	876(43.0)	985(56)	
Divorced or separated	306(8)	189(9.3)	117(7)	
Widowed	46(1)	29(1.4)	17(1)	
Infection routes				< 0.0001
PWID	99(2.6)	59(2.9)	40(2.3)	
Homosexual	2073(54.6)	968(47.5)	1105(62.9)	
Heterosexual	950(25)	557(27.3)	393(22.4)	
Others^a^	674(17.8)	455(22.3)	219(12.4)	
CD4 cell counts	205(75--287)	88(25--162)	287(245.5--330.5)	< 0.0001^#^
WHO stage				0.24^**δ**^
Stage I	15(0.4)	8(0.4)	7(0.4)	
Stage II	46(1.2)	14(0.7)	32(1.8)	
Stage III	2860(75.3)	1142(56)	1718(97.8)	
Stage IV	875(23.1)	875(42.9)	0(0)	
Time to initiation^b^ (months)	2(1--10)	1.5(1--6)	3(1.5--13)	< 0.0001^#^
Stratification of interval time				< 0.0001
≤ 3 months	2314(61)	1414(69)	900(51)	
3–12 months	674(18)	266(13)	408(23)	
> 12 months	808(21)	359(18)	449(28)	
Year of ART initiation				< 0.0001
2008	152(4)	121(5.9)	31(2)	
2009	254(6.7)	189(9.3)	65(4)	
2010	420(11.1)	257(12.6)	163(9)	
2011	601(15.8)	318(15.6)	283(16)	
2012	703(18.5)	375(18.4)	328(19)	
2013	1181(31.1)	561(27.5)	620(35)	
2014^c^	485(12.8)	218(10.7)	267(15)	

Results are shown as N (%) or the median (Q1--Q3)

*ART* combined antiretroviral therapy, *PWID* people who inject drugs

^#^By Mann-Whitney test

^a^Including transmission via plasma donation (*n* = 2), mother-to-child transmission (*n* = 3), blood transmission (*n* = 55) and undetermined transmission (*n* = 614)

^δ^This *P* value was detected by Chi-square analysis of the WHO stages I, II and stage III groups. The stage IV patients were not included

^b^Time from the diagnosis of HIV to ART initiation

^c^Comprising data only from January 2014 to June 2014

### Predictors for late ART initiation

Using a multivariate logistic regression model, we found that the patients’ age, gender, and infection routes and the calendar year of ART initiation were all independently associated with the late initiation of ART. Compared with patients younger than 30 years old, those who were 31–40, 41–50 and ≥51 years old were 1.2-, 1.6-, and 1.7-fold more likely to receive late initiation of ART, respectively. Male patients were more likely to initiate ART late than females (AOR 1.8, 95% CI 1.4–2.3). The patients who were infected by heterosexual transmission or other routes (mainly including undetermined routes) had an increased risk for late ART. Finally, compared with patients starting ART in the first half of 2014, those who initiated ART in 2008, 2009, 2010, 2011 or 2012 were more likely to be delayed for ART initiation. All of the aforementioned results were significant (*P* < 0.01). More details are listed in Table 2.

**Table 2:** Logistic regression analysis for the risk factors for late ART initiation from 2008 to 2014

Variables	Crude odds ratio (COR)	95% confidence interval (CI)	Adjusted odds ratio (AOR)	95% confidence interval (CI)	*P*-value
Age (years)					
≤ 30	Reference	--	Reference	--	--
31–40	1.33	1.13--1.56	1.20	1.01--1.43	0.044
41–50	2.00	1.66--2.42	1.58	1.27--1.97	<0.0001
≥ 51	2.28	1.89--2.74	1.68	1.34--2.12	<0.0001
Gender					
Female	Reference	--	Reference	--	--
Male	1.22	0.97--1.52	1.81	1.40--2.34	<0.0001
Marital status					
Single	Reference	--	Reference	--	--
Married or live together	1.67	1.45--1.91	1.07	0.89--1.26	0.467
Divorced, separated or widowed	1.83	1.45--2.31	1.21	0.93--1.58	0.152
Infection route					
Homosexual	Reference	--	Reference	--	--
Heterosexual	1.62	1.36--1.89	1.36	1.13--1.64	0.001
Others^a^	2.37	1.98--2.85	2.06	1.69--2.51	<0.0001
PWID	1.68	1.12--2.54	1.38	0.90--2.11	0.142
Year of ART initiation					
2014^b^	Reference	--	Reference	--	--
2013	1.11	0.90--1.37	1.16	0.94--1.45	0.175
2012	1.40	1.11--1.77	1.40	1.11--1.78	0.005
2011	1.38	1.08--1.75	1.44	1.13--1.85	0.003
2010	1.93	1.48--2.52	1.91	1.45--2.50	<0.0001
2009	3.56	2.55--4.97	3.45	2.45--4.86	<0.0001
2008	4.78	3.10--7.37	4.59	2.95--7.15	<0.0001

*ART* combined antiretroviral therapy, *PWID* people who inject drugs

^a^Including transmission via plasma donation, mother-to-child transmission, blood transmission and undetermined transmission

^b^Comprising data only from January 2014 to June 2014

### Trends in the baseline CD4 cell counts and the rate of late ART initiation over time

**Fig. 2:**
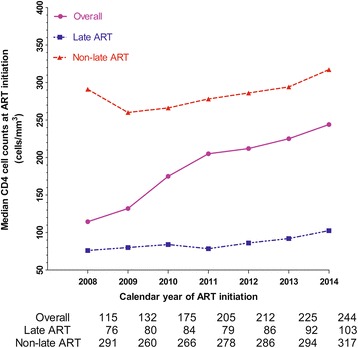
The trends of baseline median CD4 cell counts from 2008 to 2014

**Fig. 3:**
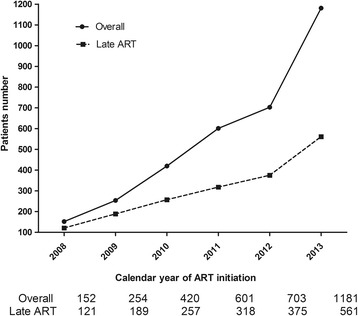
The number of overall and late ART patients over time (2008--2013)

**Fig. 4:**
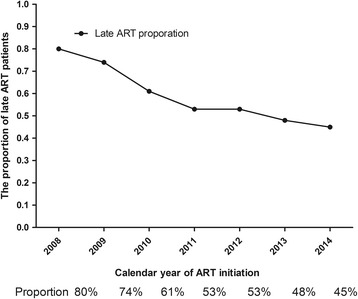
The proportion of late ART patients over time (2008--2014)

In 2008, the median CD4 cell counts among the late ART group, the non-late ART group and the overall patients were 76 cells/mm^3^, 291 cells/mm^3^ and 115 cells/mm^3^, respectively. From that time on, the CD4 cell counts of the overall patients who initiated ART increased every year. In the first half of 2014, the CD4 cell counts of the late ART group, the non-late ART group and the overall patients were 103 cells/mm^3^, 317 cells/mm^3^ and 244 cells/mm^3^, respectively. (p for trend <0.001). See Fig. 2 for more details. The number of patients initiating ART late increased slowly, while the overall number of patients initiating ART grew sharply (Fig. 3). The proportion of late ART initiation was 80% in 2008 and decreased steadily to 48% and 45% in 2013 and 2014, respectively (p for trend <0.001) (Fig. 4).

## Discussion

To our knowledge, this study is the first survey of the trends in CD4 cell counts and the first analysis of the predictors for late ART initiation of HIV-positive patients in Shanghai. In this study, we observed that the median CD4 cell counts at the initiation of ART among overall HIV patients and in the late initiation group increased steadily from 2008 to 2014 in Shanghai. At the same time, the overall number of patients who received ART increased sharply, while the number of patients initiating ART late grew slowly and the proportion of patients with late ART initiation fell rapidly.

The above observation reflects the great improvement on HIV prevention and treatment that has been made in Shanghai China for the last few years. Since 2004, China has compiled three editions of its HIV prevention and treatment guidelines, and people meeting the criteria for HIV treatment can obtain free ART medications [3, 4, 14]. Furthermore, routine CD4 cell count measurements and HIV-RNA tests are performed for free. Thus, the number of HIV-positive patients was found to be growing quickly in recently years, especially in 2013, when the guidelines expanded the treatment initiation criteria from CD4 counts <350/mm^3^ to CD4 counts <500/mm^3^ [2]. Accompanied by these changes, public education on the AIDS epidemic was expanded throughout the country, and HIV screening was also vigorously expanded [15]. Consequently, an increasing number of at-risk populations for HIV have the chance to receive an HIV diagnosis earlier than before [16]. Therefore, the CD4 cell counts of HIV patients at ART initiation have increased each year. However, the rate of late ART initiation in the first half of 2014 was still high (45%), and thus, much more effort is still needed for future prevention and treatment.

We found that male gender, older age, and heterosexual transmission were all risk factors for delayed ART. Similar to the study from sub-Saharan Africa [17] and the meta-analysis [18], compared with females, in our study the male patients were more likely to receive delayed treatment initiation. Female patients have more chances to be diagnosed early because of family planning, gynecological follow-up and prenatal screening [19]. In this study, patients older than 30 years were closer to the point of progression to advanced HIV disease when they initiated ART. This is consistent with the research data from Canada [20], Asian multicenter study [21] and Mozambique study [22]. At the same time, like the findings of Cescon A, et al. [20] and Boettiger D, et al. [21], patients who were infected via heterosexual transmission were more at risk for the delayed initiation of HIV treatment. We assumed that compared with younger patients less than 30 years old, the older patients often did not have sufficient information from the internet or the AIDS related education which is often launched in school or other semi-closed surroundings, and thus, their knowledge of HIV was limited before they progressed to AIDS. Furthermore, since the risk of acquiring of HIV among heterosexuals is lower as compared to homosexual men, most of the HIV education did not attach importance to this group, while the homosexual patients received more attention from HIV prevention agencies or NGOs (non-government organizations) and had more opportunities to obtain knowledge of HIV transmission and screening tests [6]. Thus, in contrast to the homosexual patients, the heterosexual group was more likely to initiate ART late. Therefore, both of these populations should be given more opportunity to obtain knowledge of the HIV epidemic and AIDS. Previous studies [21, 23] have shown that the people who inject drugs (PWID) are more likely to initiate ART late; however, in our study, this trend was not significant by a multivariate logistic regression test. It is possible that the number of PWID (99 patients) in our analysis was not large enough.

In comparing the late initiation group and the non-late group, one of the results should be noted: the time to initiation was significantly different between the two groups. Patients with late ART initiation had a much shorter time interval from testing positive to commencing ART. This result is reasonable, and to some extent, it implies that the late ART initiation is mainly due to the patients receiving delayed HIV diagnoses. This finding is consistent with that of previous studies [23, 24].

There are still some limitations to be considered. First, the patients’ information was collected from the clinic, and the information regarding HBV and HCV infection status, educational level, and career background was unavailable. Undoubtedly, this might skew the analysis of risk factors for delayed ART initiation. Second, we did not analyze the impact of late ART initiation on the outcome of HIV-positive patients because of the unavailability of follow-up data. However, many other studies [1, 8, 9] have demonstrated that the late initiation of ART significantly weakens its effect. Thirdly, those patients with missing CD4 cell count were excluded out of this study and it might result in selection bias.

## Conclusions

Notable improvements are observed in the median CD4 cell count at ART initiation and the proportion of patients receiving late ART initiation from 2008 to 2014 in Shanghai. However the high risk of HIV exposure persons who are male, older even heterosexual orientation should be given more education on HIV test and be encouraged to take HIV screening test in a timely fashion. This study highlights the need to improve strategies to diagnose HIV-positive individuals earlier. In this regard, expanding HIV screening programs to target the population at risk for HIV with the aforementioned predictors represents an ideal strategy.

## Additional file

Additional file 1: The demographics and clinical data of all HIV patients related to this analysis. (XLS 507 kb)

## Abbreviations

AIDS: Acquired immunodeficiency syndrome
AOR: Adjusted odds ratio
ART: Anti-retroviral therapy
CDC: Centers for disease control
CI: Confidence interval
COR: Crude odds ratio
HBV: Hepatitis B virus
HCV: Hepatitis C virus
HIV: Human immunodeficiency virus
IQR: Interquartile range
NGO: Non-government organizations
PWID: People who inject drugs
WHO: World health organization
